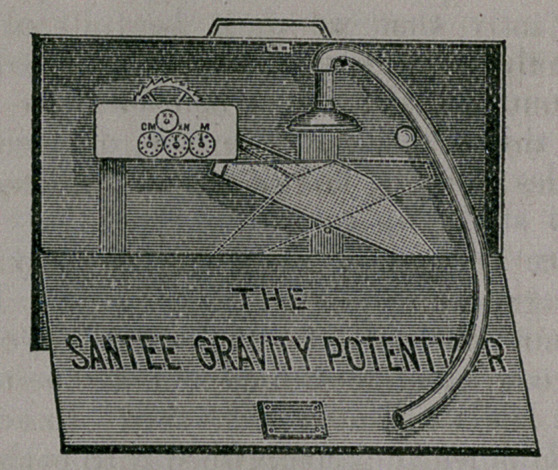# A New Potentizer

**Published:** 1889-02

**Authors:** E. B. Nash


### A NEW POTENTIZER.

While talking with my student, Dr. Ellis M. Santee, one day last summer, the subject of potencies came up. The young doctor had observed enough of my practice to become convinced that the CM potencies were wonderfully efficacious, when closely prescribed. I always try to satisfy a student upon this point, before I let him into the secret of their preparation, lest he should fall into the error of the American Institute of Homœopathy—that there is no efficacy in anything above the 12th, because they cannot discover any of the *substance* of the drug with the microscope. Of course he had read the *Organon*, and knew that Hahnemann used to recommend the 30th. That is the first book my students read.

In the conversation about potencies, I explained how the very high potencies of Swan, Fincke, Skinner, and Johnstone were made; the difference between fluxion and succussion potencies, etc.

The question finally arose, Why could not a potentizer be made that would be so cheap and simple that each physician could make *his own* potencies? This was among physicians a "long-felt want."

A full set of potencies now in the market is quite expensive for most young men beginning the practice of medicine, and if they do not possess a full set, they are very liable to want first the one they do not have. It is quite a long and tedious process to run up by hand a remedy, even to the 200th, which is not, when done, very high.

Then, again, one may have used the highest he has, and want to go higher, when he must wait until he can send for it, which he cannot often do, and thus he is obliged to lose the benefit to himself and patient, which he might get if he had a rapidly potentizing machine with which to run it up then and there.

It would be interesting to note the steps by which we came to the very perfect instrument which is now offered to the profession. The accompanying cut is very easily understood. The machine is accurately measured, making as you please, the centesimal potency on one side or the decimal on the other.

It can be set going and left, and accurately registers from the 1st to the CM.

It potentizes at the rate of about 2m per hour, but this varies according to the water present.

The tube furnishing the water for the running of the machine is easily attached to any ordinary faucet, and the stream being forced through a cap perforated with thirty fine holes, more thorough succussion is really accomplished than could be by the ordinary hand-shake of ten strokes.

The weight of the potentizing menstrum accomplishes the emptying of the cup as the required amount for each potency is added, and the side of the machine containing the medicine is immediately brought into position for refilling, to complete each succeeding potency by the same power which empties it, the water falling on the opposite side of the partition which divides the gravitating vessel.

I had thought that when this potentizer was perfect, and we should bring it the notice of the profession, I would publish cases cured by the remedies prepared by it.

But that is unnecessary, for, as the Irishman says, "the proof of the pudding is in the ateing of it."

In price it is within the reach of every one, and if physicians would potentize their own remedies (so they know what they are) and apply them in the cure of the sick, according to the rules laid down in Hahnemann's *Organon*, there would be less scoffing and more homœopathic cures.

But let no man imagine that a potency can cure, unless it has been properly selected.

*No amount of potentizing or materializing can make one un-homœopathic remedy the simillimum.*

Dr. Santee is at present in the Hahnemann Medical College of Philadelphia, where inquiries can be addressed until April 1st, after which his address will be Cortland, N. Y.

Dr. J. T. Kent has been running one of the potentizers for some time, and can add his testimony as to their merits.

CORTLAND, N. Y., Jan. 19th, 1889.

E. B. NASH, M. D.

### TESTIMONIAL.

"I have used the potentizer invented by Mr. Santee, and know its potencies to be perfectly fluxion centesimal. I have also used the potencies with proper effect."—J. T. Kent.

## Figures and Tables

**Figure f1:**